# A Two-Scale Multi-Resolution Topologically Optimized Multi-Material Design of 3D Printed Craniofacial Bone Implants

**DOI:** 10.3390/mi12020101

**Published:** 2021-01-20

**Authors:** Jaejong Park, Tareq Zobaer, Alok Sutradhar

**Affiliations:** 1 Department of Mechanical Engineering, Prairie View A&M University, Prairie View, TX 77446, USA; japark@pvamu.edu; 2 Department of Mechanical and Aerospace Engineering, The Ohio State University, Columbus, OH 43210, USA; zobaer.1@osu.edu

**Keywords:** topology optimization, craniofacial surgery, bone replacements, multi-material, bone implants

## Abstract

Bone replacement implants for craniofacial reconstruction require to provide an adequate structural foundation to withstand the physiological loading. With recent advances in 3D printing technology in place of bone grafts using autologous tissues, patient-specific additively manufactured implants are being established as suitable alternates. Since the stress distribution of these structures is complicated, efficient design techniques, such as topology optimization, can deliver optimized designs with enhanced functionality. In this work, a two-scale topology optimization approach is proposed that provides multi-material designs for both macrostructures and microstructures. In the first stage, a multi-resolution topology optimization approach is used to produce multi-material designs with maximum stiffness. Then, a microstructure with a desired property supplants the solid domain. This is beneficial for bone implant design since, in addition to imparting the desired functional property to the design, it also introduces porosity. To show the efficacy of the technique, four different large craniofacial defects due to maxillectomy are considered, and their respective implant designs with multi-materials are shown. These designs show good potential in developing patient-specific optimized designs suitable for additive manufacturing.

## 1. Introduction

The main function of the skeletal system is to provide a stable foundation for body support and to facilitate human movement [1], with bones being the primary components of this system. Each bone has unique geometry, and bones come in different sizes to properly serve adequate purposes in their own environment [2]. A surgical procedure to include bone replacements is usually necessary when this skeletal system is damaged. However, this process becomes complicated if the defect site is large or located near the region where functionality like biting as well as aesthetic appearance are significant factors (e.g., midface) [3]. These bone defects usually require bone replacement and autografts; allografts and metal-based bone implants have traditionally been preferred. In the case of midface reconstruction, free flaps including fibula or iliac crest are widely used for autografts due to their reliability. Metal-based implants in bone reconstruction surgeries also have a long history. The efforts to use Titanium as implant materials date back to 1930 [4]. Through decades of research efforts, it has been established that Titanium and its alloys are more suitable than other metal alloys for bone implants due to their superior biocompatibility, bioactivity, and mechanical properties [5]. Another advantage of Titanium-based materials compared to bone grafting is that they provide limited geometric freedom since they are usually machined from sheets or billets. Multi-axis CNC centers allow machining of complex geometries and provide an excellent surface finish. Yet, realizing intricate internal geometries like cancelleous bone is often difficult and time-consuming due to machining limitations [6].

With a recent surge in 3D printing technologies, reconstruction surgeries are having a paradigm shift with the potential of creating patient-specific solutions. It significantly decreases the speed of part delivery since it avoids complex tasks associated with the machining such as CNC programming, tooling, etc. [7]. More importantly, it allows the manufacture of patient-specific complex geometry due to its additive nature, laying down very thin layer of material at a time to create a complete 3D structure. Thus, the structures with intricate internal features can be easily realized. Novel strategies that take advantage of additional freedom in geometry quickly became one of the core research focus in the medical field and many patents have been treated since early 1990s as demonstrated in [8,9]. More recently, 3D printing has been employed in tissue engineering to fabricate small size scaffolds to treat periodontal defects [10]. 3D printed biosensors have demonstrated their efficacy, low-cost, and sensitive biomedical diagnostics [11]. Multidisciplinary studies have proposed different computer-based design algorithms for bone implant geometry. In [12], symmetric mandible implants were designed based on patient images. Various reverse engineering-based methods have been developed to enhance the macroscopic shape fidelity enhancement implants for defective skulls [13]. A porous patient-specific spinal interbody fusion implant was designed in CAD and 3D printed with Titanium for shortened procedure time and improved fusion in [14]. Custom CAD-designed and 3D printed with PCL composite airway splint significantly increase the survival rate from tracheomalacia in animal models [15] as well as human object [16]. Computer engineered wavy patterns in scaffolds have also shown to enhance osteogenesis in human Mesenchymal Stem Cells (hMSC) [17]. In [18], bone density and anisotropy of human proximal femur were predicted using homogenization. A macroscale spinal interbody cage with polyaryletheretherketone (PEEK) titanium and cortical materials to address stress shielding is shown in [19].

**Figure 1:**
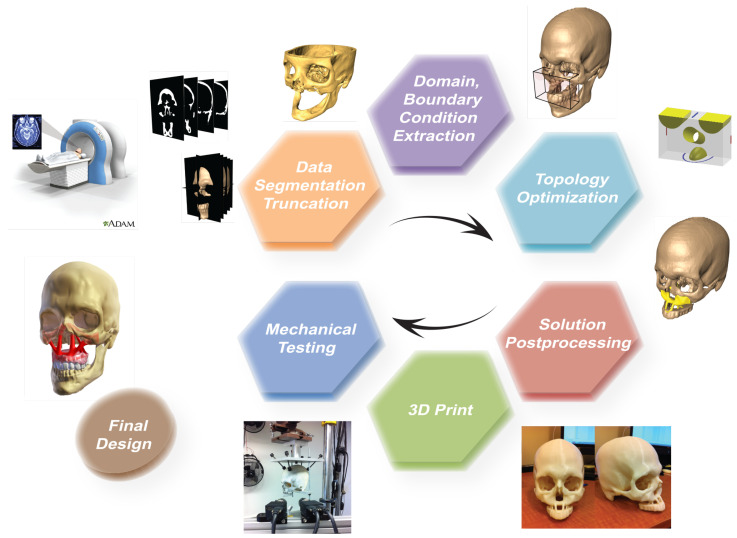
The workflow of using structural topology optimization for craniofacial bone replacement.

Structural optimization tools, more specifically topology optimization, can be employed to understand the relationship between, geometry, and function of bone and bone-implant by targeting the optimal performance measures. We previously developed a method that uses topology optimization to design bone replacements for craniofacial reconstruction [20], which follows the work flow illustrated in Figure 1. Using this technique, it was shown in [20] that the generic human craniofacial bone structure can be attained when proper load and boundary conditions are utilized in topology optimization. The method was implemented in a variety of clinical cases, which revealed potential in reviving efficient load transfer mechanisms [21]. An experimental validation using digital image correlation on a 3D printed skull based on the topology optimized bone replacement implants was presented in [22].

Topology optimization has taken great strides due to its synergy with advancements in 3D printing [23]. Multi-material structure is one of the fast-evolving fields in 3D printing. This is particularly appealing to the medical industry as it significantly broadens the design options for a wide variety of implants for efficient and controlled healing [24]. Complex functional requirements such as bone implant design for craniofacial reconstruction can be tackled with multi-material topology optimization for a potential enhanced outcome. There have been multiple research efforts in the multi-material topology optimization framework. Unique material properties with different volume fractions are required in multi-material topology optimization. In this setting, material interpolation schemes that would make the potential material combinations satisfy the Hashin–Shitrikman bounds become crucial. Bendsoe and Sigmund proposed Solid-Isotropic material with Penalization (SIMP)-like formulations for any element with two materials without vanishing stiffness [25]. Extensions to three-phase structures (two material with a void phase) are presented to design microstructures with extreme properties in [26], and the recursive method allows interpolation of three or more material selections [27]. Hvejsel and Lund [28] unified SIMP and Rational Approximation of Material Properties (RAMP). Gaussian distribution is used in [29] to weight each phase, and the material is assigned when the design variable coincides with a material peak function. In [30], a multi-material scheme that considers nonlinear material behavior is introduced with an update scheme that optimizes design variables with its volume constraint only, thereby improving the efficiency of optimization. Implicit function-based methods for multi-material topology optimization include color level set [31], phase field methods using the Allen–Cahn system [32], or Cahn–Hilliard model [33], among others.

In this work, we present a two-scale topology optimization to design a multi-material bone implant with consideration of optimized microstructures. This study aims to explore a computer-aided algorithm for designing optimized multi-material bone implants with functional property (e.g., stiffness, specific functionalities, etc.) control. In the first stage, topology optimization will distribute different materials at hand in an optimal way to obtain the best properties macroscopically. The second stage is based on replacing the solid material with a microstructure selected from a library of microstructures, where each of them is designed for different elastic properties using topology optimization. The outcome is a 3D printable structure that will be porous due to the presence of holes and internal geometries in the microstructures. In contrast, the macrostructure in the first stage will be a combination of multiple topologies, each representing distribution of materials with different material properties.

The remainder of paper is structured as follows: Section 2 introduces the topology optimization formulation utilized in the first and second stage and further describes the concept, implementation, and aspects of both the multi-material macrostructures and microstructure design. Section 3 details the craniofacial bone replacement cases with different types of defects that are going to be addressed in the paper. The optimized solutions are illustrated and discussed in Section 4 which were obtained for the defects using topology optimization; finally, the conclusion is in Section 5.

## 2. Methods

### 2.1. Stage 1: Multi-Material Bone Implant Macrostructure Using Multi-Resolution Topology Optimization

In this section, methods used to obtain the macrostructure are presented. The macrostructure will efficiently consider multiple material options through topology optimization. The mathematical formulation for multi-material problem is described. Inherently, additional material consideration promotes complexity in the problem, which consequently increases the computation time. A multi-resolution approach is implemented in the multi-material topology optimization to reduce the time spent in computing the structural response.

#### 2.1.1. Multi-Material Topology Optimization

Topology optimization iteratively reaches the solution by modifying the number, size, and shape of the voids simultaneously in the design domain. In density element methods, topology optimization is formulated as a material distribution problem. A usual formulation of topology optimization minimizes the compliance. Here, the formulation distributes a limited amount of material such that the final topology is the stiffest (minimum energy of deformation) with respect to the input boundary conditions, i.e., supports and loadings. The minimum compliance formulation for multi-material topology optimization is considered in the present work. In a design domain $\Omega \in R^{2}$ or $R^{3}$, a multi-material topology optimization finds optimal locations of a *m* number of different materials that minimize the compliance under a volume fraction constraint $V_{s}$ (amount of material that can be utilized by the algorithm). Finite elements that discretize the design domain are used to evaluate the performance and visualize the optimum solution. The normalized density, ρ (or ρ when combined into a vector), is the design variable. Since one element can have combinations of different materials in multi-material topology optimization, densities at a given location are represented with an array of density values for different material phases $\rho_{i}$(i=1,…,m). The problem is written as,

(1) $$\begin{array}{ccc} \min_{\rho }C\left(\rho_{i},u\right) & = & u^{T}Ku\\ s.t.:K(\rho_{i})u & = & f\\ \int_{\Omega }\rho_{i}d\Omega & \leq & V_{i}\;\mathrm{where}\;\left(i=1,\ldots ,m\right), \end{array}$$

where *C* is the compliance, $\rho_{i}$ is the density vector of material phase *i*, f defines the global loads, and global displacements are represented by u. K is the global stiffness matrix, and $V_{i}$ is the volume fraction allowed for *i*th material phase.

Allowing density to have intermediate value requires a material interpolation scheme to obtain reasonable local material properties. SIMP [25] and the homogenization method [34] are two of the more popular schemes. As an example for single material topology optimization, the SIMP method penalizes intermediate density values to parameterize local material properties in the following way,

(2) $$E\left(\rho \right)=\rho^{p}E^{0},$$

where, $E^{0}$ is the elastic modulus for a specific material in the solid phase (ρ = 1) and the *p* is the penalization factor. Most of the multi-material topology optimization works focus on the standard or modified versions of the aforementioned SIMP formulation. In [35], materials with different properties were treated using ordered SIMP. Combinatorial SIMP method for multi-material structures suitable for polyjet 3D printing was presented in [36]. Following the original three-phase interpolation scheme in [26], Chan et al. [37] proposed a general material interpolation scheme that allows any arbitrary number of materials. This interpolation scheme is used design multi-material lattice structures against uncertainties in material and load. ZPR, another design update scheme, can handle an arbitrary number of design variables [38]. ZPR updates design variables by only considering its associated volume constraint and the corresponding Lagrangian multiplier. In the present work, the interpolation scheme described in our earlier work [39] is used for efficiently distributing multiple material phases in the design domain, which is given by,

(3) $$E\left(\rho \right)=\sum_{i=1}^{m}\rho_{i}^{p}E_{i}^{0}.$$

Here, $E_{i}^{0}$ is the elastic modulus for phase *i* in solid phase ($\rho_{i}=1$). For a multi-material problem, Equation (3) essentially expresses elastic modulus at a point as sum of all *m* elastic moduli present at that point. It is important to note that this interpolation scheme may violate Hashin–Shtrikman bounds in cases where $\rho_{i}$ is low, and *p* is high [40]. In the multi-material topology optimization formulation adopted in this work, the problem is divided into several two phase material distribution problems, and they are solved sequentially in each cycle, in a Gauss–Seidel like algorithm [32]. Thus, in each of the subproblems, the two phases ‘*a*’ and ‘*b*’ having densities equal to $\rho_{a}$ and $\rho_{b}$, respectively, are distributed optimally, while keeping densities of all other phases constant. Mathematically, at each location *x*,

(4) $$\rho_{a}+\rho_{b}=1-\sum_{i=1,i\neq a,b}^{m}\rho_{i}\left(x\right).$$

The subproblem is further simplified by taking density of one phase, $\rho_{a}$ as the design variable, since $\rho_{b}$ can be readily calculated using Equation (4).

#### 2.1.2. Multi-Resolution Topology Optimization

The resolution from the result of traditional element-based topology optimization, for instance, problem formulated in Equation (1), is based on the discretization of the design domain. Longer computational time is inevitable if a solution with high resolution is desired. In this work, a computationally efficient multi-material topology optimization is performed by using multiple levels of the mesh. This multi-resolution approach provides results in finer resolution than the base design domain discretization. A coarse discretization is used in the displacement mesh for the system equilibrium, and relatively finer meshes are used for the design variable and the density element [41]. Different meshes communicate with each other via a projection function, which also promotes mesh-independent solution, and avoids checkerboard [42]. Topology optimization formulation in Equation (1) is rewritten below to accommodate the features of the multi-resolution method,

(5) $$\begin{array}{ccc} \min_{\rho }C\left(\rho_{i},u\right) & = & u^{T}Ku\\ s.t.:K(\rho_{i})u & = & f\\ \rho & = & f_{p}\left(d\right)\\ \int_{\Omega }\rho_{i}d\Omega & \leq & V_{i}\;\mathrm{where}\;\left(i=1,\ldots ,m\right). \end{array}$$

Here, d is the design variable vector, and $f_{p}\left(.\right)$ is the projection function. To benefit from the multi-resolution method, a density mesh needs to be finer than the displacement mesh. For the multi-material problem, the elemental stiffness considers contribution from all residing materials within the displacement element, which is mathematically expressed as,

(6) $$K_{e}\left(\rho_{e}\right)=\sum_{n=1}^{N_{n}}\;\left(\int_{\Omega_{e/n}}B^{T}(\sum_{i=1}^{m}{\rho_{n}}_{i}^{p}D_{i}^{0})B\;d\Omega_{e/n}\right).$$

In Equation (6), ${\rho_{n}}_{i}$ is the amount of the *i*th phase of the density element *n* in the displacement element *e*, $N_{n}$ is the total number of density elements within a displacement element, B is the matrix of shape function derivatives, and $D_{i}^{0}$ describe the mechanical behavior of phase i when it is in the solid phase (${\rho_{n}}_{i}=1$). We use 3D MTOP elements following [41] where B8/n125 element represents an 8-noded brick element with 125 embedded density element. A simple linear filter with the efficient optimality criteria is used for updating the design variables in each iteration. Derivatives of the objective function and constraints are required for this design update. They are given by,

(7) $$\begin{array}{cc} \frac{\partial C}{\partial d_{N}} & =-u_{e}^{T}\frac{\partial K_{e}}{\partial d_{N}}u_{e} \end{array}$$

(8) $$\begin{array}{cc} \frac{\partial V}{\partial d_{N}} & =\sum_{n=1}^{N_{n}}\frac{\partial V}{\partial \rho_{n_{a}}}\frac{\partial \rho_{n_{a}}}{\partial d_{N}} \end{array}$$

Here, $d_{N}$ is the design variable (that corresponds to phase ‘*a*’) in each binary phase (i.e., distributing phase ‘*a*’ and ‘*b*’) subproblem, $u_{e}$ the displacement vector of displacement element *e*, and $\rho_{n_{a}}$ denotes the density of phase ‘*a*’ inside density element *n*. Derivative of the stiffness matrix, $K_{e}$, is calculated as,

(9) $$\frac{\partial K_{e}}{\partial d_{N}}=\sum_{n=1}^{N_{n}}\frac{\partial K_{e}}{\partial \rho_{n_{a}}}\frac{\partial \rho_{n_{a}}}{\partial d_{N}}=\sum_{n=1}^{N_{n}}p{\left(\rho_{a}\right)}^{p-1}\cdot \int_{\Omega_{e/n}}B^{T}\left(D_{a}^{0}-D_{b}^{0}\right)Bd\Omega_{e/n}\frac{\partial \rho_{n_{a}}}{\partial d_{N}}$$

Readers are referred to [39] for more details regarding the sensitivity analysis.

### 2.2. Stage 2: Bone Implant Internal Geometries and Functional Microstructures

Porous bone implants have many benefits that include structural weight reduction, which in turn affects the mechanical behavior of the bone implant, and also providing a larger surface area for bone healing. Utilizing microstructural variation also offers to control mechanical characteristics. This is especially important as most of the materials used to fabricate the bone implant are stronger than the bone itself, leading to non-uniform stress distribution and affecting long-term reliability [43]. In the present work, the structures are first optimized by distributing solid materials in the design domain. Then, they are replaced by porous materials of a desired property. Two methods for implementing porosity are discussed in this section. Perimeter control allows arranging structural fibers (internal structures) in an optimal way by providing efficient load transfer paths. Tailoring the microstructure is another way to impose porosity in the structure with architected material properties. The formulations of inverse homogenization in topology optimization are also briefly discussed.

#### 2.2.1. Perimeter Control

Perimeter is defined by the sum of inner and outer boundaries. In a 2-dimensional design domain with an arbitrary density distribution, the perimeter P(ρ) can be computed by the following equation,

(10) $$\begin{array}{c} P\left(\rho \right)=\sum_{j=0}^{X}\sum_{i=1}^{Y}l\cdot |\rho_{i,j}-\rho_{i-1,j}|+\sum_{i=0}^{Y}\sum_{j=1}^{X}l\cdot \left|\rho_{i,j}-\rho_{i,j-1}\right|, \end{array}$$

where *l* is the edge length of an element and X×Y represents the mesh size of the design domain. The minimum value on a perimeter can be defined to divide large void regions into smaller ones, potentially providing thinner structural members in the final design. Thus, an additional constraint is prescribed in the optimization problem as,

(11) $$\begin{array}{c} P\geq P_{min}, \end{array}$$

where $P_{min}$ is the minimum perimeter value allowed in the final solution. A traditional filter is still required to regulate the common numerical instabilities such as checkerboard pattern. The following linear filter is employed in this work,

(12) $$\tilde{\rho_{e}}=\frac{\sum_{\Omega_{e}}w_{ne}\cdot \rho_{e}}{\sum_{\Omega_{e}}w_{ne}}.$$

Here, $\tilde{\rho_{e}}$ is the filtered density value, and $w_{ne}=max\left(0,r_{min}-dist\left(n,e\right)\right)$ is defined as the distance function between the center of the filter (*n*) to elements (*e*) within the zone of filter influence $\Omega_{e}$ having a radius of $r_{min}$. For handling multiple constraints, an optimizer like Method of Moving Asymptotes (MMA) [44] is typically used. From the numerical examples in [43], it was observed that when linear filtering and perimeter control are used for topology optimization with regular patterns in the initial design domain (including uniform density distribution), stable results are not obtained. The numerical instabilities can be significantly decreased by introducing randomness into the initial design domain, as shown in [43]. In addition, the structure retained natural-looking topological characteristics, making it feasible to mimic the trabecular arrangement of human bone. In [43], we demonstrated the scheme for mimicking the internal geometry of the human proximal femur.

#### 2.2.2. Microstructure Design

The coveted mechanical behavior in the macroscale can also be obtained by manipulating the microstructure with the macrostructure. Homogenization allows to mathematically relate the effective property of the macroscale to that of microscale. However, the homogenization approach assumes that the microstructure is periodically repeated throughout the macrostructure, and there is a significant scale difference between micro and macro level [40]. If *x* and *y* are macroscopic and microscopic variables, respectively, and ϵ is the ratio of micro- to macroscale length, then, within a *Y*-periodic unit cell (i.e., single microstructure) asymptotic expansion of the total displacement field, $u^{\epsilon }$, leads to the following relation,

(13) $$\begin{array}{c} u^{\epsilon }=u^{0}\left(x\right)+\epsilon u^{1}\left(x,y\right) \end{array}$$

Here, $u^{0}\left(x\right)$ is the macro-level displacement and $u^{1}\left(x,y\right)$ is the *Y*-periodic microscopic fluctuation from $u^{0}\left(x\right)$. Consequently, a problem can be established such that topology optimization designs a microstructure for desired macroscale properties by minimizing the difference between homogenized effective stiffness tensor and the target stiffness tensor; this is commonly called the inverse homogenization. The homogenized stiffness tensor in an unit cell of area *Y* is obtained from the following equation,

(14) $$\begin{array}{c} E_{ijkl}^{H}=\frac{1}{\left|Y\right|}\int_{Y}\left(E_{ijkl}-E_{ijpq}\frac{\partial \chi_{p}^{kl}}{\partial y_{q}}\right)dy, \end{array}$$

where, $\chi_{p}^{kl}$ are *Y*-periodic test fields found by solving the following equilibrium equation,

(15) $$\begin{array}{c} \int_{Y}E_{ijpq}\frac{\partial \chi_{p}^{kl}}{\partial y_{q}}\frac{\partial \phi_{i}}{\partial y_{j}}dy=\int_{Y}E_{ijkl}\frac{\partial \phi_{i}}{\partial y_{j}}dy\;\;\;\;\;\forall Y-\mathrm{periodic}\;\;\phi . \end{array}$$

**Figure 2:**
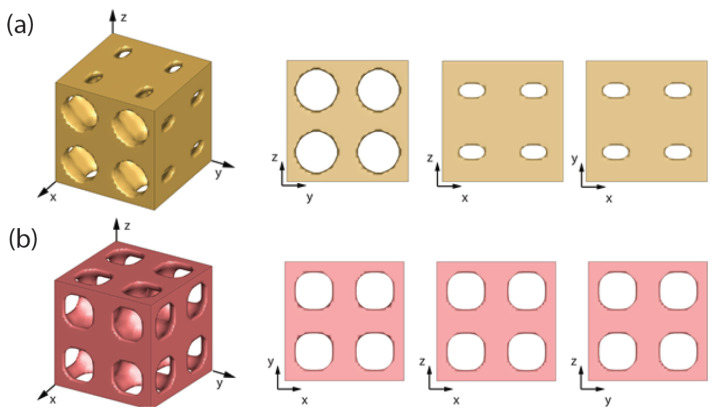
Design of microstructure using topology optimization. Maximization of (**a**) uniaxial (*x*) stiffness, and (**b**) bulk modulus.

In three dimensions, the optimization problem for inverse homogenization can be written as,

(16) $$\begin{array}{c} \min_{\rho }\sum_{i,j,k,l=1}^{3}\omega_{ijkl}{\left(E_{ijkl}^{*}-E_{ijkl}^{H}\left(\rho \right)\right)}^{2}\\ s.t.:K\chi^{kl}=f^{kl}\\ V\leq V_{s}\\ 0<\rho \leq 1, \end{array}$$

where the design variable ρ is the density of element which can have any value between 0 and 1, $\omega_{ijkl}$ are weight factors that control the importance of a component of the squared $L_{2}$-norm between the target and homogenized effective stiffness tensor, $E^{*}$ and $E^{H}$, respectively, in the objective function, and $V_{s}$ is the prescribed volume fraction constraint. Equation (15) is solved using the finite element method in Equation (16), where *K* is the global stiffness matrix, $f^{kl}$ is the force vector, and $\chi^{kl}$ is the characteristic displacement of the unit cell due to unit test strain in the kl direction. Possible combinations of kl requires three normal (k=l) and three shear (k≠l) strains tests. Readers are directed to [40] for a detailed description of mathematical derivation. Inverse homogenization has been widely employed to design microstructures having a desired structural property. For example, the microstructure for maximizing the uniaxial stiffness ($E_{1111}$) can be obtained using this technique as shown in Figure 2a. The following equation relates bulk modulus with the elasticity tensor,

(17) $$\begin{array}{c} K=\frac{1}{9}\left(E_{1111}+E_{2222}+E_{3333}+E_{1122}+E_{1133}+E_{2211}+E_{2233}+E_{3311}+E_{3322}\right), \end{array}$$

which can be used to obtain the microstructure for maximum bulk modulus (*K*), as shown in Figure 2b.

## 3. Craniofacial Structure and Segmental Defects

**Figure 3:**
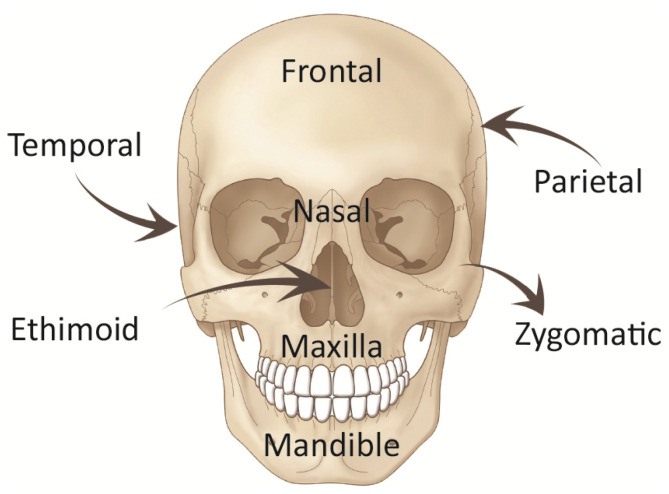
Basic facial skeleton anatomy.

The human facial skeleton has the mandible (jawbone) at the bottom, and ethmoid, vomer, while frontal bone constitute the midline cranium that encases the brain. Figure 3 depicts the anatomy of the facial skeleton. The bones in the midface need to maintain the conspicuous nasal cavity for breathing, hold up the orbit, and transfer the loads from daily human activities (e.g., mastication). Three distinct buttress systems [45,46] exist in the craniofacial structure through which the loads are spread and relieve the stress. Therefore, craniofacial reconstruction should ensure the recovery of these structure pillars after maxillectomy to preserve a strong foundation for basic functional needs.

When a massive portion of the bone is injured or missing, it is termed as segmental defect. Cordeiro and Santamaria [47] classified maxillary defects based on the extent of maxilla resection. Limited maxillectomy (Type I) is the defect where the palate is intact, but a few maxilla walls have been resected. Subtotal maxillectomy (Type II) is where the lower five walls are resected with the preserved orbital floor. If all six walls of the maxilla and partial orbit are removed, it is categorized as a total maxillectomy (Type III). Different bone implants need to be implemented to provide the foundation for the eyeball and reconstruct lost buttress systems in this case. Finally, a patient is diagnosed with the orbitomaxillectomy (Type IV) if the hard palate is intact with five maxilla walls and portions of the orbital floor are resected.

**Figure 4:**
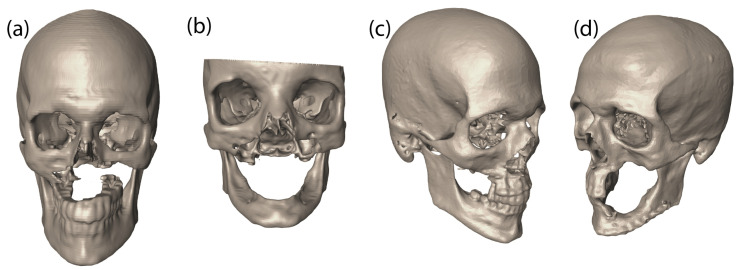
The segmented Digital Imaging and Communications in Medicine (DICOM) data with an isotropic spatial resolution of the four clinical cases used in this study are shown. The DICOM data have a total of 512 images on the frontal and sagittal planes. The number of images on the horizontal plane varies depending on each case. (**a**) Case 1: Bilateral subtotal maxillectomy. (**b**) Case 2: Bilateral subtotal maxillectomy II. (**c**) Case 3: Right limited maxillectomy. (**d**) Case 4: Left limited maxillectomy and mandibular defect in left lateral segment.

In a previous work [21], we studied the feasibility of using the bone replacement shapes by creating 3D printed models and fusing them in a 3d printed skull with defect. Mechanical testing and finite element results indicated that the bone replacement shapes obtained via single material topology optimization not only withstand maximum mastication force, they can also restore adequate load transfer mechanism in the midface [22,48]. In the present work, we perform multi-material topology optimization to design bone replacement shapes for four patient-specific craniofacial defects created by cancer resection. Having metal bone implants can induce stress-shielding and other undesirable effects. Hence, optimal shapes of bone replacements with different material properties in different regions are obtained from multi-material topology optimization for the following four different cases in Figure 4.

The segmented image data of the four clinical cases are shown in Figure 4. The defects in Cases 1 and 2 lie in the middle of the midface and bilaterally extend asymmetrically, which led to a complete loss of a lower portion of the maxilla, thus partially separating teeth from the craniofacial skeleton. The bone replacement for these two cases needs to provide a bony structure that can support the loadings from daily oral activities. The defect in Case 3 is small and limited to the right side of alveolar processes. The bone replacement for this defect requires a structural platform for dental implants which will aid in a balanced mastication. Case 4 is a left limited maxillectomy, which also misses a lateral segment of the mandible. Two different bone replacements are necessary for a midfacial and mandibular defect to restore normal structural functions of the midface.

## 4. Results and Discussion

Bone grafts are often required to recover the structural integrity in the midface in case of a large bone loss. Improper treatment may result in patient dissatisfaction or failure in the long run. The image is segmented from the patient Digital Imaging and Communications in Medicine (DICOM) data to show the facial skeleton. The appropriate size of the design domain is acquired by using a measuring tool. Since the multi-material topology optimization approach does not carry any units during the process, it is the ratio of the 3D Cartesian axial lengths that are input to the algorithm. Acquired numbers from the measurement are carefully rounded (so that the shape of the actual design domain is as close to the measured defect region) into integers which essentially represent the dimensions of the design domain of the topology optimization in their respective directions. Patient-specific load and boundary conditions are extracted using the unique design domain in the defect region whereas the volume fraction is judiciously selected so that it would match with that of the undamaged bone. The efficient multi-material topology optimization is used to design patient-specific bone replacement shapes. For simplicity, all examples consider two material phases and a void phase. A fixed stiffness ratio of 3 with a Poisson’s ratio of 0.3 are used for the two material phases. Single material bone replacement shapes are provided with their corresponding multi-material topology optimization results for visual comparison. Following the standard practice for showing any 3D results, all designs in this section are visualized by drawing density iso-surfaces with 25% of the maximum density value for all phases. The multi-resolution element we used in this work is a B8/n125 MTOP element; 125 density elements exist in a single 8-noded brick element.

### 4.1. Stage 1: Multi-Material Bone Implant Macrostructure

#### 4.1.1. Case 1: Bilateral Subtotal Maxillectomy I

**Figure 5:**
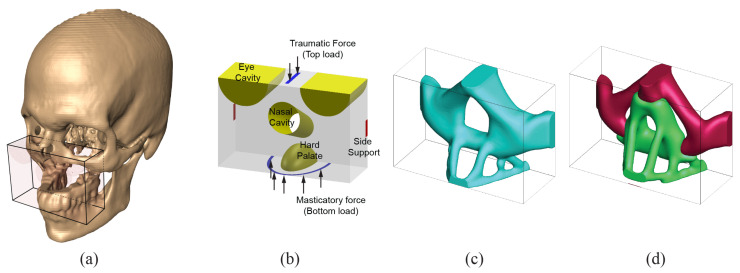
Case 1. (**a**) Design domain extraction from patient CT scan, (**b**) boundary condition used in the analysis, (**c**) bone replacement shape using single material topology optimization, (**d**) bone replacement shape using multi-material (2 materials) topology optimization.

The domain of Case 1 is shown in Figure 5a and the size is chosen as 28 (width) × 20 (height) × 12 (depth) for the topology optimization. Unit forces (purely upwards) that follow dentition which simulates the loads from mastication activity are applied on the bottom face, and a group of unit forces in the opposite direction is placed on the top surface, mimicking possible traumatic forces that may be transmitted from the neurocranium. These loading and support boundary conditions are adopted from our previous work [20]. Since the design domain covers nasal passageway, hard palate, and a portion of eye orbits, prescribed voids are embedded to ensure that similar features are obtained in the multi-material topology optimization result. To allow an adequate contact and fixation to the uninjured portion of the facial skeleton, side supports are defined on the two lateral faces, as shown in Figure 5b.

**Figure 6:**
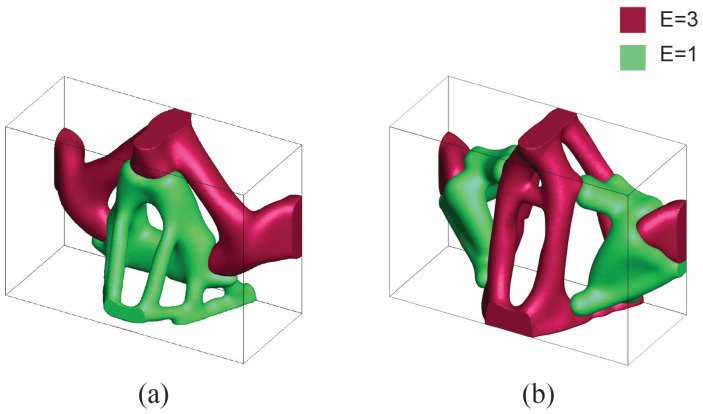
Effect of the ratio of traumatic force (**top**) and the masticatory force on the design for Case 1. (**a**) **top**/**bottom** = 10, (**b**) **top**/**bottom** = 1.

The chosen domain is discretized into a total of 6720 (W28×H20×D12) B8/n125 elements. Volume fraction constraint ($V_{s}$) is selected as 12% and penalization factor (*p*) of 3 is selected. The size of the minimum length scale ($r_{min}$) is equal to the length of a displacement element. This volume fraction constraint is chosen by comparing the volume occupied by the actual bone geometry of the defect region from the clean reference skull with the volume of the design domain. The load ratio between the traumatic force (top) and the masticatory force (bottom) is 10, and the number of iteration is limited to 50. Single material topology optimization is shown in Figure 5b for comparison. For the multi-material analysis, two different material phases with Young’s moduli values of 3 and 1 are used. Void phase is modeled with $E=10^{-9}$. Volume fractions for each phases are 6% for the stiff material (E=3), 6% for the soft material (E=1) and 88% for the void. The multi-material result is shown in Figure 5d. One can verify that all the prescribed voids for facial features are well described in single and multi-material solutions. Compared to the single material topology optimization result, the overall shape is fairly similar without any significant differences. The stiff material is assigned to form a structure that would transfer the top traumatic forces to the supports in the lateral surfaces. In contrast, most of the softer material is assigned near the mastication loading and the nasal cavity area. This is expected, as top traumatic loading is ten times more significant than the bottom mastication loadings. A completely different solution is obtained if equal importance between the top and bottom loadings is assumed, as shown in Figure 6b. If the top/bottom load ratio is changed from 10 to 1, one can observe that in Figure 6b a significant amount of stiff material is situated to the lower portion of the design domain to withstand the large load.

#### 4.1.2. Case 2: Bilateral Subtotal Maxillectomy II

**Figure 7:**
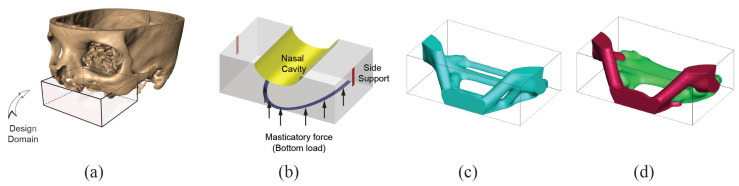
Case 2—Scenario 1: mastication loading is purely vertical. (**a**) Design domain extraction from patient CT scan, (**b**) boundary condition used in the analysis, (**c**) bone replacement shape using single material topology optimization, (**d**) bone replacement shape with 2 material topology optimization.

**Figure 8:**
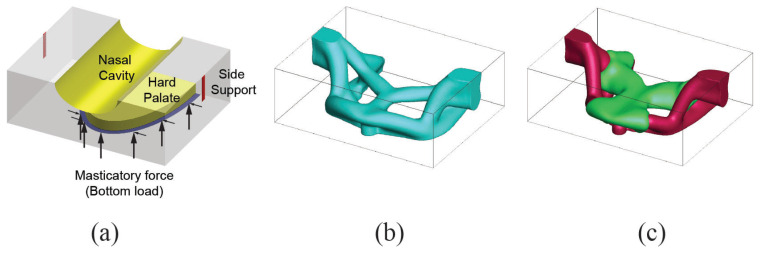
Case 2—Scenario 2: mastication loading has 2 components vertical and horizontal towards center. (**a**) Boundary condition used in the analysis, (**b**) bone replacement shape using single material topology optimization, (**c**) bone replacement shape using multi-material (2 materials) topology optimization.

The defect in Case 2 is smaller but similar to Case 1. In this scenario, traumatic forces are omitted in the design consideration because the uninjured portion of the maxilla is thicker and the defect is fairly symmetric compared to Case 1. Two different scenarios are considered based on whether the mastication loading is purely vertical or not. The domain size is discretized by W32×H11×D20 for pure vertical masticatory loading (Scenario 1), whereas the design domain for the skewed mastication loading (Scenario 2) is slightly elongated in depth-wise direction to be W32×H11×D25. Skewed mastication loading in Scenario 2 adds a small horizontal component that is 20% of a vertical component towards the domain center. Supports are provided in both the lateral surfaces. The nasal cavity and are hard palate are the voids introduced in the analysis. The size of the minimum length scale ($r_{min}$) and the penalization factor remain the same as Case 1 for both scenarios. The 15% and 10% volume fraction constraints are employed for Scenarios 1 and 2, respectively. For the multi-material analysis, volume fraction constraints are equally divided for the two material phases. Design domains with respective boundary conditions are illustrated in Figure 7 and Figure 8.

In both scenarios, results from multi-material topology optimization conserve the overall shape from single material topology optimization. Stiff material forms a structure that encompasses the structure with soft materials. Typically, we observed that the stiff material is assigned in critical areas where loads and supports are located.

#### 4.1.3. Case 3: Right Limited Maxillectomy

**Figure 9:**
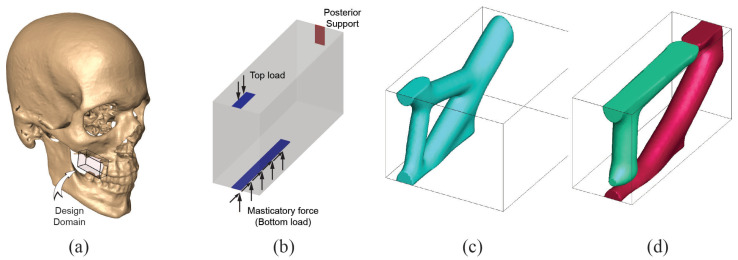
Case 3. (**a**) Extraction of design domain from patient CT scan, (**b**) boundary condition used in the analysis, (**c**) bone replacement shape using single material topology optimization, (**d**) bone replacement shape using multi-material (2 materials) topology optimization.

The defect in Case 3 is small compared to previous cases and is from right limited maxillectomy. The surgery left a defect in the alveolar processes in the molar teeth and the posterior maxilla. The bone replacement will also serve as a platform for the dental implant. From the measurement, the domain size is chosen to be W6×H9×D17. Supports are provided on the back surface to ensure a structural member for proper fixation, and the masticatory force is simulated on the bottom surface towards the anterior face. This loading has both the vertical (upward) and the horizontal (toward posterior face) components with a ratio of 1 between them. A purely vertical force (downward) is placed on the top face to provide a vertical load transfer path. Volume fractions for stiff (E=3) and soft (E=1) are equally 5%. Penalization factor is kept at 3, and minimum length scale is again equal to the length of a displacement element. Design domain and boundary conditions are given in Figure 9.

Resulting optimized structures are shown in Figure 9c,d. The horizontal force component in the bottom load led the solution to have a thick and direct connection (member) between their locations to the support at the back surface. From this thick member, the loading from the top surface is linked in a different way for single and multi-material topology optimization. In addition, it is interesting to note that the masticatory forces from the bottom (which is more significant than the top forces due to the higher net force magnitude) have taken stiffer material, whereas softer material is assigned to handle less significant loadings from the top.

#### 4.1.4. Case 4: Left Limited Maxillectomy and Mandibular Defect in Left Lateral Segment

**Figure 10:**
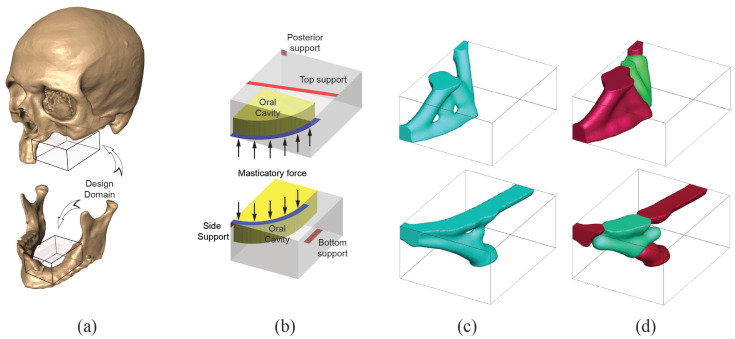
Case 4. (**a**) Design domain extraction from patient CT scan, (**b**) boundary condition used in the analysis, (**c**) bone replacement shapes using single material topology optimization, (**d**) bone replacement shapes using two material topology optimization.

Case 4 requires two different bone replacement shapes for the maxilla and mandible. Both bone replacement shapes need to withstand the loading from daily human activity and to provide a stable mechanism to transfer the load. Two separate design domains are considered to address these structural requirements. The domains are measured to be W15×H7×D18 for the maxilla (top) and W12×H7×D15 for the mandible (bottom) (see Figure 10a). Purely vertical (upward for the top and downward for the bottom) forces are applied to the dentition. Suitable oral cavities are also introduced to not disturb oral activities when implemented in the defect region. Supports are added to ensure secure attachment to the uninjured portion of facial skeleton as shown in Figure 10b. Penalization factor is selected as 3 with 10% for volume fraction constrain (5% each for stiff and soft for multi-material analysis). The minimum length scale remains the same as in previous cases. Bone replacement shapes using single material and multi-material topology optimization are presented in Figure 10.

**Figure 11:**
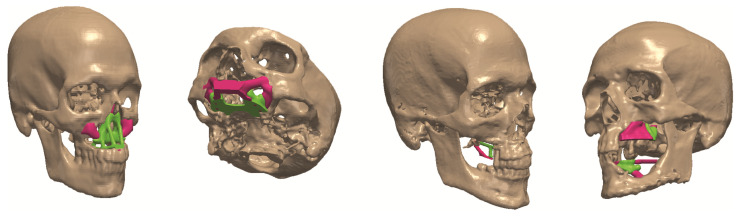
Bone replacements embedded in the defect.

Finally, the final topologies of the craniofacial region with the inserted bone replacements are shown in Figure 11.

### 4.2. Stage 2: Bone Implant Internal Geometries and Functional Microstructures

**Figure 12:**
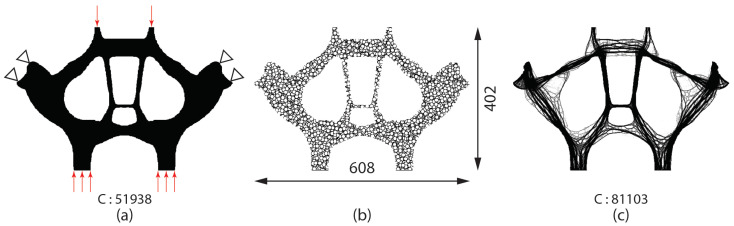
Geometric complexity control using perimeter control constraint [43]. (**a**) Cross-section of midface across first molar teeth, (**b**) initial configuration generated by randomly locating circles in the design domain (*P* = 46,187), (**c**) topology optimized internal geometry ($P_{min}$ = 30,000). A 60% increase in the compliance is observed while achieving 80% volume (weight) reduction.

To further improve the structural efficiency or reduce the overall weight of the craniofacial implant, porosity can be introduced to the overall structure by designing the internal geometry of solid elements. Additional geometric constraints, for example the perimeter control described in Section 2.2.1, can be added to the problem. In our earlier work [43], we demonstrated the geometric complexity of the cross-section in the midface implant using the perimeter control approach. The cross-section is taken from the first molar, which is discretized with 608×402 Q4 linear elements. Active design domain is patterned with circles with 5 units in radius in random locations to provide initial perimeter of 46,187. With a lower perimeter bound of 30,000, and volume fraction of 20%, the internal geometries evolved to have more fibrous structural members, as shown in Figure 12.

**Figure 13:**
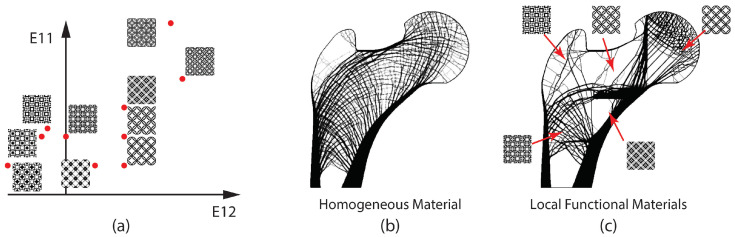
The effect of microscopic property variation for trabecular architecture using perimeter control. (**a**) Microstructure library for different macroscopic properties $E_{11}$ and $E_{12}$ represents axial and shear properties, respectively, (**b**) reference design using homogeneous material, and (**c**) a sample design using local functional materials with different macroscopic properties.

Structural topology optimization with inverse homogenization has shown great potential in obtaining microstructures for desired mechanical properties in macroscale. In designing large scale bone replacements for a wide variety of reconstruction surgeries, this approach can be used to control local mechanical properties. This can avoid abrupt differences in the structural behavior at the interface between the bone and the implant, improving the implant longevity and osseointegration. The structures illustrated in Figure 13a are the microstructures that manifest different properties when stacked in a large number. The vertical axis represents the relative axial strength, whereas the horizontal axis represents the relative shear properties. These microstructures serve as a material library to manipulate the local mechanical behaviors in the macrostructure. Utilizing these microstructures with various properties leads to a complete geometric configuration in the macroscale. Structure in Figure 13b is topology optimized with a perimeter control constraint in the femur design domain. Here, typical homogeneous material is used, and the overall structure resembles the trabecular architecture of the human proximal femur. A completely different structure can be obtained by utilizing various microstructures, as shown in Figure 13c. Homogenization-based algorithms, such as void morphing [49], can be employed to ensure smooth interface connectivity between different regions of microstructures, which is important for a good structural performance.

Bone undergoes a continuous remodeling process to enhance its homeostasis [50]. Depending on its density, two distinct bone regions exist: cortical bone and cancellous bone. Denser cortical bone takes approximately 80% of bone mass supporting most of the heavy loads caused by ambulation in the case of long bones in the human leg. The cancellous bone also serves a structural role by transferring the articular surface load to the cortical bone [51]. The design of trabecular configuration in the human femoral head is shown in Figure 13b. Functionally graded materials are beneficial in modeling such materials whose mechanical properties vary by location in the computational design. The placement of stiffer microstructures in highly loaded areas such as primary compressive and tensile group and softer microstructures in the Ward triangle in the femoral head will provide an option to create more 3D printing friendly implant designs.

**Figure 14:**
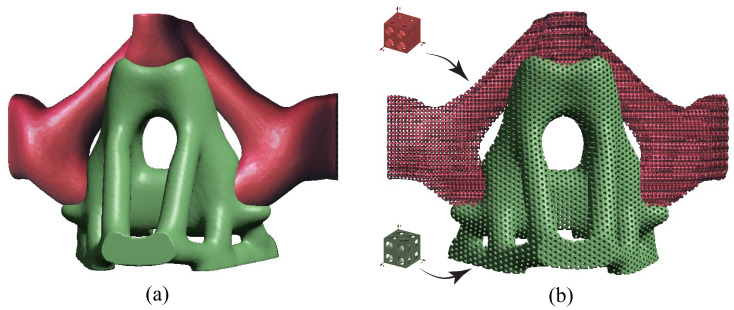
(**a**) Stage 1 multi-material topology optimization of the macrostructure for Case 1, (**b**) Stage 2 multi-material topology optimization with microstructure presenting two different materials for Case 1.

Based on the desired material properties, we obtain the microstructure pattern for two different materials for the Case I problem. Then, they are used to replace solid material phases in the design. In Figure 14, both the macrostructure and the microstructure from the two-stage optimization are presented. Here, the pattern of unit cells designed to maximize uniaxial stiffness and bulk modulus replaced each homogeneous material phase, respectively.

## 5. Conclusions

We presented a topology optimization approach that provides multi-material designs for bone replacement implants in complicated craniofacial segmental defects. In this approach, the macroscale design provides the optimized load transfer mechanism providing the maximum stiffness, while the microstructure design provides material properties that are required in different regions of the implants. These techniques have the potential to offer efficient 3D printed bone implants. Using a multi-material method for bone replacement is appropriate since the materials required for manufacturing these implants are typically expensive. The topology optimization procedure presented here blends a multi-resolution approach with a multi-material topology optimization technique. Designing adequate bone replacements for segmental defects is a critical problem that deals with different important aspects. The quantitative analysis of bone replacements may require consideration of (i) mechanical variables such as structural integrity during load-bearing, (ii) biological concerns for instance vascularization for healing, and (iii) functional considerations such as creating passageways for respiratory airflow and transit of food and liquid from the mouth to the pharynx. Each of these must be accomplished while preserving normal human appearance. Such variables can be included in a multi-scale topology optimization framework, which seeks the optimal layout of the reconstructed craniofacial region. Innovative scaffold for bone growth may be engineered with multi-materials by taking advantage of composite materials with recent developments in multi-material 3D printers. Patient-specific simulation and virtual planning of the interventions using sophisticated numerical modeling and advanced 3D printing (also rapid prototyping, stereolithographic modeling) can enhance the optimization of the treatment and improve the quality of life for the patient.
